# Carpal tunnel syndrome due to an atypical deep soft tissue leiomyoma: The risk of misdiagnosis and mismanagement

**DOI:** 10.1186/1477-7819-5-92

**Published:** 2007-08-08

**Authors:** Byron E Chalidis, Christos G Dimitriou

**Affiliations:** 1 1^st ^Orthopaedic Department of Aristotle University of Thessaloniki, Greece; 2 Department of Orthopaedic Surgery "Hippokration" General Hospital, Thessaloniki, Greece

## Abstract

**Background:**

Leiomyomas of the deep soft tissue are quite uncommon and occur even more rarely in upper extremity.

**Case presentation:**

A 32-year old manual laborer man presented with a two-year history of numbness, tingling and burning pain in the palmar surface of the left hand and fingers. His medical history was unremarkable and no trauma episode was reported. According to the clinical examination and the result of median nerve conduction study (NCS) the diagnosis of carpal tunnel syndrome was established. Operative release of the transverse carpal ligament was subsequently performed but the patient experienced only temporary relief of his symptoms. MRI examination revealed a deep palmary located mass with well-defined margins and ovoid shape. Intraoperatively, the tumor was in continuity with the flexor digitorum superficialis tendon of the middle finger causing substantial compression to median nerve. Histopathological findings of the resected mass were consistent with leiomyoma. After two years the patient was pain-free without signs of tumor recurrence.

**Conclusion:**

Despite the fact that reports on deep soft tissue leiomyoma are exceptional, this tumor had to be considered as differential diagnosis in painful non-traumatic hand syndromes especially in young patients.

## Background

Leiomyomas are uncommon smooth muscle tumors and they account for 4.4% of all benign soft tissue neoplasms [1]. They are subdivided into two major groups based predominantly on location: superficial and deep soft tissue leiomyomas [2]. Deep soft tissue leiomyomas are often present as larger masses and can show a wide spectrum of histological changes [3]. They tend to be larger than those of superficial counterparts, probably because they produce few symptoms and therefore may not be detected until a relatively later stage [2].

We describe an unusual case of secondary carpal tunnel syndrome due to a misdiagnosed, deep-seated, soft tissue leiomyoma. Surgical excision led to uneventful clinical result and resolution of symptoms.

## Case presentation

A 32-year old manual laborer, right-hand-dominant man presented in the Orthopaedic Outpatient Clinic of the local hospital with a two-year history of numbness and tingling sensation in the palmar surface of left hand and fingers. During the last six months the patient reported also burning pain in the same area – causing him to wake up frequently during the nightime hours – and progressive inability to perform his regular occupation. No episode of trauma or other medical problems were mentioned. All the symptoms were attributed to carpal tunnel syndrome and the diagnosis was confirmed with median nerve conduction study (NCS). Through a classical open palmar approach subsequent release of the transverse carpal ligament was performed. According to surgeon's operative report, there wasn't any reference for any pathology in the dissected area except from the severely compressed median nerve. However, postoperatively, the patient experienced only partial and temporary relief of his symptoms without improvement of hand function.

**Figure 1:**
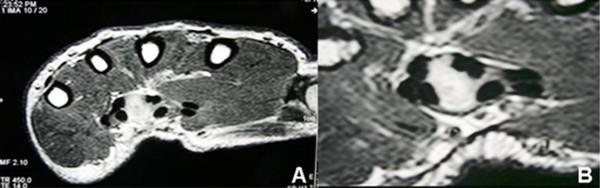
**A**. MRI (T1-weighted image) of the left hand showing a soft tissue mass into the carpal tunnel **B**. Under higher magnification, the tumor looks homogenous, well-defined and it is surrounded by the flexor tendons.

Three months after the surgical procedure, the patient was referred to our department for further investigation. On physical examination, the palmar aspect of the left hand and the area of the previous incision were tender to palpation and swollen while a slight atrophy of the thenar muscles was apparent. There was a sensory loss in the area innervated from the median nerve as compared with the controlateral hand and Tinel's sign was evoked over the median nerve at the wrist. The patient had decreased grip strength while passive flexion and extension of fingers were painful. As a result, additional investigation was deemed necessary and magnetic resonance imaging (MRI) was selected for delineating any potential pathology in the carpal tunnel or the surrounding tissues. MRI showed a misdiagnosed, volarly placed soft tissue mass with well-defined margins and an ovoid shape which was in direct contact with the flexor tendons (Figure 1).

**Figure 2:**
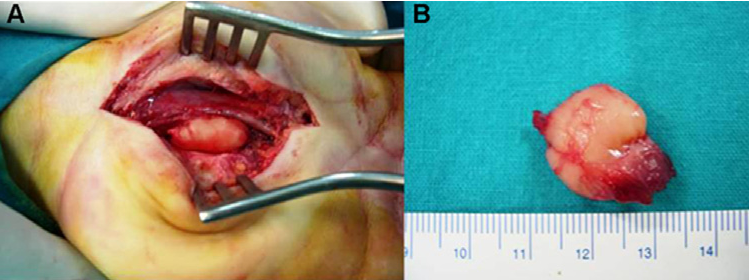
**A**. Intraoperative image of the tumor. Significant compression and macroscopic changes of median nerve are visible **B**. Macroscopic appearance of the resected tumor. Its diameter is approximately 1.5 cm.

**Figure 3:**
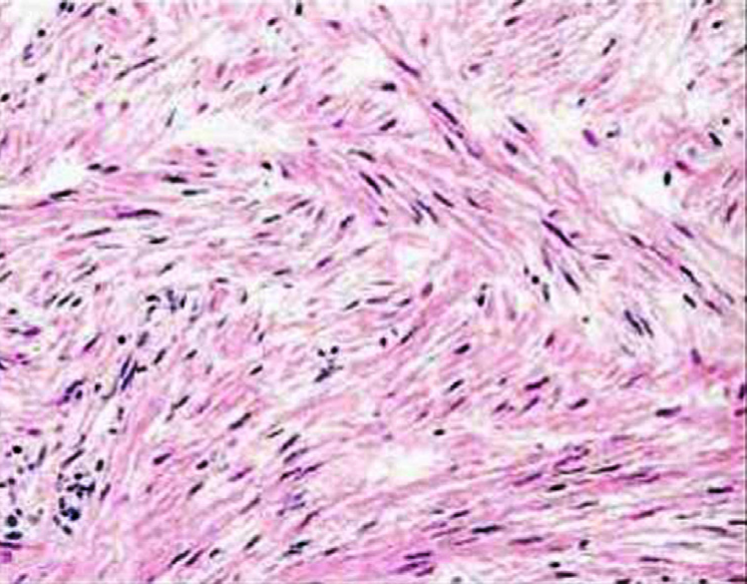
Microscopic appearance of the tumor. Bundles of spindle-shaped cells arranged in interlacing fascicles with elongated, oblong nuclei and eosinophilic cytoplasm. There is no evidence of necrosis, pleomorphism, mitoses or nuclear atypia.

Because of the persistent symptoms and the radiographic abnormalities, surgical exploration and tumor resection were scheduled. Under axillary block a well-circumscribed, encapsulated mass in the deep palmar tissues was identified causing significant compression of the median nerve. The tumor was adhered to the third flexor digitorum tendon superficialis and it was moving within the tendon during flexion or extension of the middle finger (Figure 2) (See additional file 1). No vascularity or connection with the deep volar arch was evident. The mass was completely removed from the adjacent tissue and the histological examination of the surgical specimen revealed spindleshaped cells arranged in fascicles with cigar-shaped nuclei and eosinophilic cytoplasm. The smooth muscle nature of the tumor was confirmed immunohistologically by a strong reactivity against smooth muscle actin (SMA) and a negative reaction against S-100 and epithelial membrane antigen (EMA). No significant cellular pleomorphism or mitotic activity was noted. The above findings were compatible with leiomyoma (Figure 3).

At the two-year follow-up evaluation, the patient showed no evidence of recurrence of the mass and no pain or sensory dysfunction. He had a significant improvement in hand grip strength and he returned to his previous occupation.

## Discussion

Leiomyomas are benign soft tissue tumors that occur wherever smooth muscle is present. They are classified into three groups: cutaneous leiomyomas that arise from the erector pili muscle; vascular leiomyomas that arise from smooth muscle of the vein and leiomyomas of deep soft tissues [3]. In hand, the tumor is exceedingly rare, it is usually located superficially and in most of the cases is vascular [4-6]. Characteristic morphologic features included well-defined circumscription with a fibrous pseudocapsule, myxohyaline stromal degeneration, and intersecting fascicles of spindle cells with mostly uniform, round-ended, elongated nuclei and tapering, eosinophilic cytoplasm [7].

Deep soft tissue leiomyomas are uncommon and only sporadic cases have been reported in the literature [1-3,8,9]. Misumi et al [2] reviewed 21 cases of deep soft tissue leiomyoma from the English literature and they found to occur at almost any age, ranging from 3 to 62 years (mean, 25 years) and more frequently affected males (14 cases) than females (7 cases). Almost half of the cases were located in the extremities (10 cases), and there was only one report with multiple deep soft tissue leiomyomas [10].

The tumor may not be easily recognised until become painful and it is rarely diagnosed before surgery as imaging techniques, including MRI, are not specific for tumor diagnosis [11]. Lipomas, leioymyosarcomas, schwannoma or neurofibroma, haemangioma, nodular fascitis, soft tissue giant cell tumor such as pigmented villonodular synovitis or giant cell tumor of tendon sheath have to be considered in the differential diagnosis [3,12]. Atypical locations of leiomyomas in hand, such as peripheral nerves or tendons may further hamper early and accurate preoperative assessment leading to clinical misdiagnosis and subsequent surgical mismanagement [13].

In the herein case, patient symptoms were attributed to idiopathic carpal tunnel syndrome and no further investigation was taken place. Even during the open dissection of the carpal tunnel, no pathology around median nerve was suspected and no meticulous observation of the area was performed. As space occupying lesions were found only in 5.5% of the patients with unilateral carpal tunnel syndrome routine imaging for the investigation of unilateral carpal tunnel syndrome is not essential [14]. However, the importance of proper clinical and radiologic assessment should not be underestimated as it was clearly dictated in the presented case. We believe that in cases of compression neuropathy in young people, a concern should be raised regarding the presence of an underlying tumor or tumorous condition especially when residual symptoms exist after initial treatment. Furthermore, surgeon should always inspect carpal tunnel and median nerve to rule out any space occupying lesion.

Typically, leiomyoma lacks mitotic activity altogether and the usual treatment is simple excision of the tumor due to its benign character. Billings et al [15] were reported neither recurrences nor metastases after excision of somatic soft tissue leiomyomas during a mean follow-up of 58.7 months with the longest follow-up being 97 months. This is even more impressive considering that five patients were known to have microscopically positive margins. However, Herren et al [7] and Neviaser and Newman [8] described malignant formation of a finger and forearm leiomyoma respectively in which further surgical procedures were required. Despite the rarity of the above phenomenon, these tumors should be approached with caution until histopathologic examination confirms the absence of nuclear atypia, necrosis and mitotic activity.

## Conclusion

The case is presented due to its rarity and the risk of tumor misdiagnosis. Deep soft tissue leiomyoma is an uncommon tumor and its appearance in upper limb or hand constitutes a rare issue. The potential of tumor connection to flexor tendons and the development of compression neuropathy make the diagnosis even more difficult as the mass may be mistaken for a giant cell tumor of the tendon sheath or a ganglion cyst. Further awareness of the existence of atypical leiomyomas in hand among clinicians may lead to better evaluation and understanding of similar soft tissue lesions.

## Competing interests

The author(s) declare that they have no competing interests.

## Authors' contributions

BC: Preparation and submission of manuscript.

CD: Data collection, preparation of figures and video.

Both authors have read the final manuscript and agree to its publication.

## Supplementary Material

Additional file 1 Adhesion of tumor to the third flexor digitorum tendon superficialis. Click here for file
